# Piezoelectric Implant Site Preparation: Influence of Handpiece Movements on Temperature Elevation

**DOI:** 10.3390/ma13184072

**Published:** 2020-09-14

**Authors:** Luca Lamazza, Marco Lollobrigida, Iole Vozza, Luigi Palmieri, Claudio Stacchi, Teresa Lombardi, Alberto De Biase

**Affiliations:** 1 Department of Oral and Maxillofacial Sciences, Sapienza University of Rome, 00161 Rome, Italy; luca.lamazza@uniroma1.it (L.L.); iole.vozza@uniroma1.it (I.V.); alberto.debiase@uniroma1.it (A.D.B.); 2 Department of Cardiovascular, Endocrine-metabolic Diseases and Aging, Istituto Superiore di Sanità, 00161 Rome, Italy; luigi.palmieri@iss.it; 3 Department of Medical, Surgical and Health Sciences, University of Trieste, 34129 Trieste, Italy; claudio@stacchi.it; 4 Department of Health Sciences, University of “Magna Græcia”, 88100 Catanzaro, Italy; drteresalombardi@libero.it

**Keywords:** piezoelectric surgery, implant site preparation, heat generation, temperature rise, working movements

## Abstract

Piezoelectric devices are widely used in oral surgical procedures, including implant site preparation. However, little is known about the influence of working movement on temperature elevation in bone. The aim of this study was to assess the effects of two different working cycles on temperature elevation during piezoelectric implant site preparation. Sixty osteotomies at a depth of 10 mm were performed on bone blocks of bovine ribs using a piezoelectric tip with external irrigation (IM1s, Mectron Medical Technology, Carasco, Italy). A mechanical positioning device was used to guarantee reproducible working and measuring conditions. Two different working cycles, of 4 and 6 s, respectively, were tested, including both longitudinal and rotational movements. Temperature was recorded in real time with a fiber optic thermometer and applied pressure was maintained under 150 g. For each test, the highest recorded temperature (T_max_) and the mean temperature recorded from 30 s before to 30 s after the highest recorded temperature (T_±30_) were extrapolated. Tests duration was also recorded. Both T_max_ and T_±30_ were significantly higher in the ‘6 s cycles’ group than the ‘4 s cycles’ group (42.44 ± 7.3 °C vs. 37.24 ± 4.6 °C, *p* = 0.002; 37.24 ± 4.6 °C vs. 33.30 ± 3.3 °C, *p* = 0.003). Test duration was also significantly higher using 6 s cycles compared to 4 s cycles (143.17 ± 29.4 s vs. 119.80 ± 36.4 s, *p* = 0.002). The results of this study indicate that working cycles of 4 s effectively reduce heat generation and working time during piezoelectric implant site preparation.

## 1. Introduction

Implant site preparation can be considered one key element for the long-term clinical success of implant rehabilitations. Different surgical procedures are described in the literature for implant site development, including conventional drilling, osseodensification, and piezoelectric techniques. Among these techniques, piezoelectric implant site preparation (PISP) represents a valuable alternative particularly in challenging anatomic conditions, as well as improving the primary stability of implants [1,2]. Piezoelectric osteotomies are characterized by a micrometric and selective cut, which means that the ultrasonic tip selectively cuts bone while sparing surrounding soft tissues [3]. It has also been reported that the ultrasonic bone stimulation could further enhance osseointegration [4,5,6]. On the other hand, some studies reported a higher risk of overheating using ultrasonic techniques compared to rotating instruments [7,8], even if adopted methodology and standardization had been questioned [9].

Heat generation during implant site preparation is a complex phenomenon that involves different factors. These can be classified into three main groups: technique-related (i.e., ultrasonic power, tip design, irrigation rate, and modality), operator-related (i.e., applied load, working movements), and bone-related factors (i.e., cortical thickness, bone density, and vascularization). Among these variables, operator-related factors are likely to be the hardest to investigate and standardize. In order to minimize temperature elevation, rapid operative movements and light pressure are suggested [10], mostly as an empirical rule since no study has investigated the influence of handpiece movements on heat generation.

The importance of motion pattern has been highlighted for the rotating technique [11]. In PISP, the effects of the handpiece movements, however, may be even more pronounced due to the heat generated inside the tip from internal friction, in addition to that generated by contact with the bone substrate. Moreover, operative movements are difficult to standardize with ultrasonic devices since they are specific for each tip.

The aim of this ex vivo study was to analyze the impact of the handpiece movements on temperature elevation during piezoelectric implant site preparation, thus identifying an ideal protocol for the safe use of piezoelectric devices in the clinical practice.

## 2. Experimental Section

**Figure 1:**
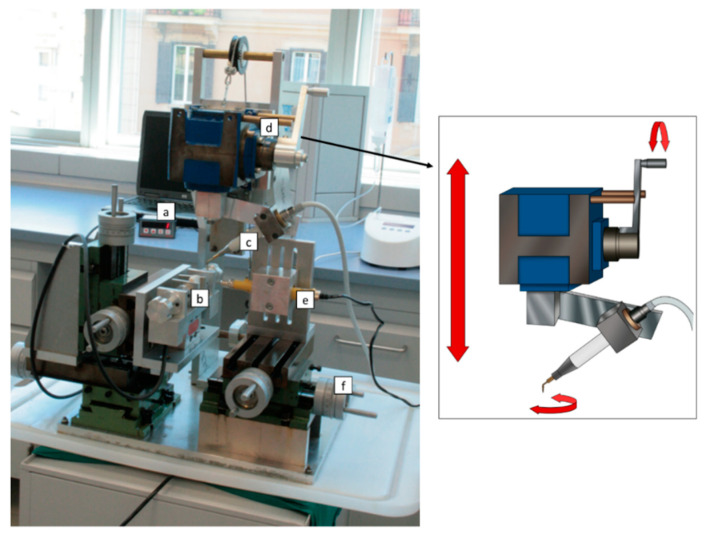
Mechanical positioning device used for the study with graphical representation of the transmission tool responsible for the handpiece movements. (**a**) Load cell, (**b**) bone sample, (**c**) piezoelectric handpiece, (**d**) transmission tool, (**e**) drill used to create the hole for the thermometer probe, and (**f**) micrometer screws to obtain three-dimensional movements of both the bone sample and the drill used to create the holes for the probe.

The study specimens consisted of blocks of bovine ribs, measured approximately 6x4x3 cm. Bovine ribs represent a well-documented bone model that resembles the human jaw due to similarities in bone density, the ratio between cortical and cancellous bone, and thermal conductivity [12,13]. In particular, it was demonstrated that, compared to other animal, human, or artificial bone specimens, bovine rib shows significantly higher temperature elevations during drilling procedure [13], thus reducing the risk of underestimating the thermal effects of bone instrumentation in the clinical practice. According to Sedlin and Hirsch guidelines [14], the bone samples were kept wet at all times, and frozen-stored in saline at −10 °C. Sixty osteotomies were performed on the specimens at a depth of 10 mm using a dedicated mechanical device (Figure 1), specifically designed to maintain constant working parameters, as described in a previous study [15].

**Figure 2:**
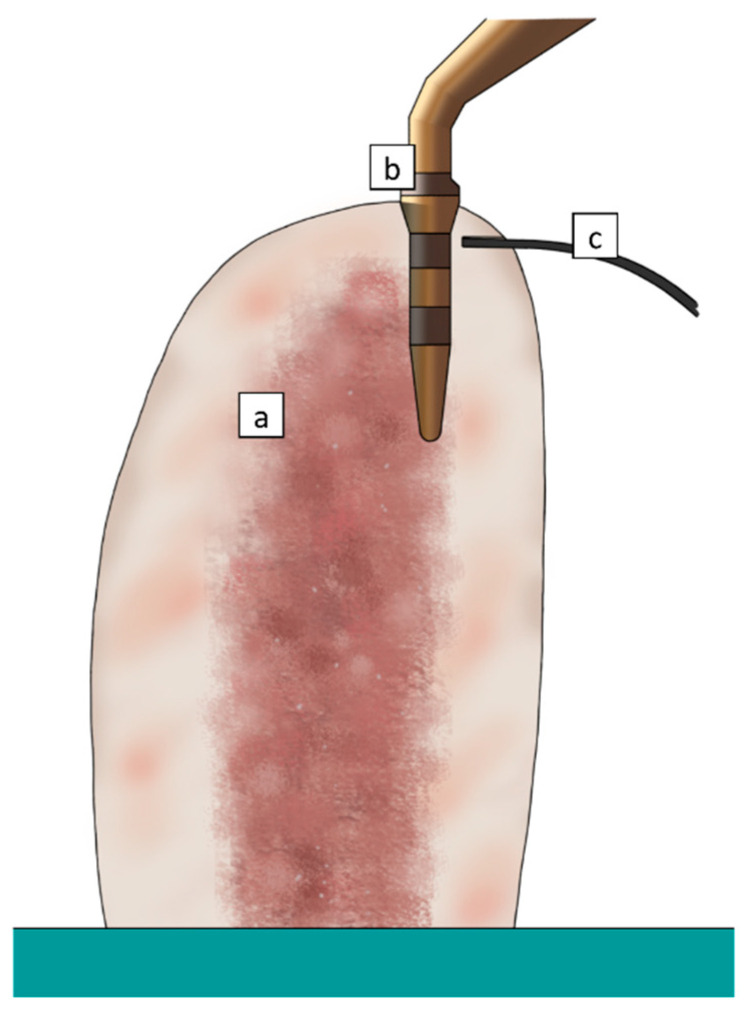
Schematic section of the bone sample (**a**) with the ultrasonic tip (**b**) and the thermometer probe (**c**) at completion of osteotomy.

Briefly, the handpiece (Piezosurgery 3^®^, Mectron Medical Technology, Carasco, Italy) was anchored to a transmission tool and equipped with a handle, allowing for both vertical and rotational manual movements. Temperatures were recorded in real time using a fiber optic thermometer (Luxtron m 3300 Biomedical Lab Kit, Luxtron Corporation, Santa Clara, CA, USA). The use of a fluoroptic thermometer for measuring temperature rise during bone instrumentation has been extensively described in a previous study [16]. This technology is based on a temperature-sensitive phosphorescent sensor included in the probing end of a fiber optic cable (0.5 mm diameter). Unlike other devices, fluoroptic thermometers are immune to electromagnetic or radiofrequency noise, and do not require a calibration procedure. The detection point was set at 0.5 mm from the tip surface, 2 mm below the top of the specimen (Figure 2). A load cell showed the real time load applied on the bone. All the tests were performed by a single expert operator maintaining the applied pressure under 150 g. The experiments were conducted at a controlled room temperature of 20–25 °C.

The handpiece motion pattern (working cycle) was a combination of three movements: longitudinal downward, rotational, and longitudinal upward. For the study purpose, the osteotomies were performed with working cycles of two different time durations, of 4 and 6 s, respectively. In the 4 s cycles the duration of each movement was: 1 s longitudinal downward, 1 s rotational, and 2 s longitudinal upward; in the 6 s cycles: 2 s longitudinal downward, 2 s rotational, and 2 s longitudinal upward. A digital metronome was used to follow this specific sequence.

A diamond-coated conical tip (IM1s, Mectron Medical Technology, Carasco, Italy), with external irrigation, was used for the tests. Among the tips used in PISP, IM1s was selected as a model due to the higher temperatures that it generates compared to the other tips. The piezoelectric unit was set in bone mode with special level power for IM1s tip. A cooling saline solution at room temperature (20–25 °C) with a flow rate of 28 mL/min was used.

For each osteotomy, the following parameters were recorded:

- Duration (s): Time necessary to perform each osteotomy to a 10 mm depth;
- Cycles: Number of cycles necessary to perform each osteotomy to a 10 mm depth;
- T_max_ (°C): Highest recorded temperature;
- T_max_ (°C): Highest recorded temperature;
- T_±30_ (°C): Arithmetic mean of all temperature data points recorded from 30 s before to 30 s after the highest recorded temperature.

In order to investigate the differences in temperatures reached in the two groups of experiments carried out with 4 s and 6 s cycles, respectively, means, standard deviations, and medians were elaborated for each group. Correlation analysis was performed among variables duration, cycles, T_max_, and T_±30_.

Given the low number of samples, the non-parametric Mann–Whitney U test for independent samples was performed to compare means of duration, cycles, and temperature between the two groups of experiments, the median test was used for comparing medians in the two groups, and Levene’s test using F-Fisher values was used for comparison of variances in the two experiment groups.

The size of 30 osteotomies for each experimental group assures a statistical power over 80% in the comparison of the mean values of duration, cycles, and temperature between the two independent groups of experiments under the hypothesis that variables were normally distributed, given the standard deviations and the differences calculated in each group, and given the Type 1 error probability of 0.05 associated with the null hypothesis that the population means of the two groups were equal.

## 3. Results

For specific variables of duration, cycles, T_max_ and T_±30_ means, standard deviations, and medians were reported by experiment group in Table 1 and Table 2 together with non-parametric tests results for means and medians comparison.

Means results were significantly higher in the ‘6 s cycles’ group than in the ‘4 s cycles’ one, for duration (143.17 s. vs. 119.80 s.; *p* = 0.002), T_max_ (42.44 °C vs. 37.24 °C; *p* = 0.002), and T_±30_ (37.01 °C vs. 33.30 °C; *p* = 0.003), while means results were significantly lower in the ‘6 s cycles’ group than in the ‘4 s cycles’ one for cycles (23.80 vs. 29.95; *p* = 0.005).

Similar results were found for medians, with statistically significant higher values in the ‘6 s cycles’ group than in the ‘4 s cycles’ one for duration (142.50 s. vs. 108.67 s.; *p* < 0.0001), T_max_ (40.71 °C vs. 37.82 °C; *p* = 0.02), and T_±30_ (36.32 °C vs. 32.94 °C; *p* = 0.02), while means resulted significantly lower in the ‘6 s cycles’ group than in ‘4 s cycles’ one for cycles (23.75 vs. 27.17; *p* = 0.02).

The F-Fisher test for comparison of variances in the two experiment groups results were statistically significant for cycles (F = 4.351; *p* = 0.041) confirming a different variability in the two groups (Table 3).

A high positive correlation was demonstrated between maximum temperature and mean temperature (ρ = 0.891), and between duration and cycles (ρ = 0.647), indicating that these two couples of variables provide the same information. However, while duration presents a similar positive correlation with T_max_ (ρ = 0.102) and T_±30_ (ρ = 0.180) even though it is not statistically significant (*p* = 0.437 and *p* = 0.168, respectively), cycles present an opposite correlation with T_max_ and T_±30_ (ρ = −0.157 and ρ = −0.041, respectively), which is also not statistically significant (*p* = 0.231 and *p* = 0.756, respectively).

**Figure 3:**
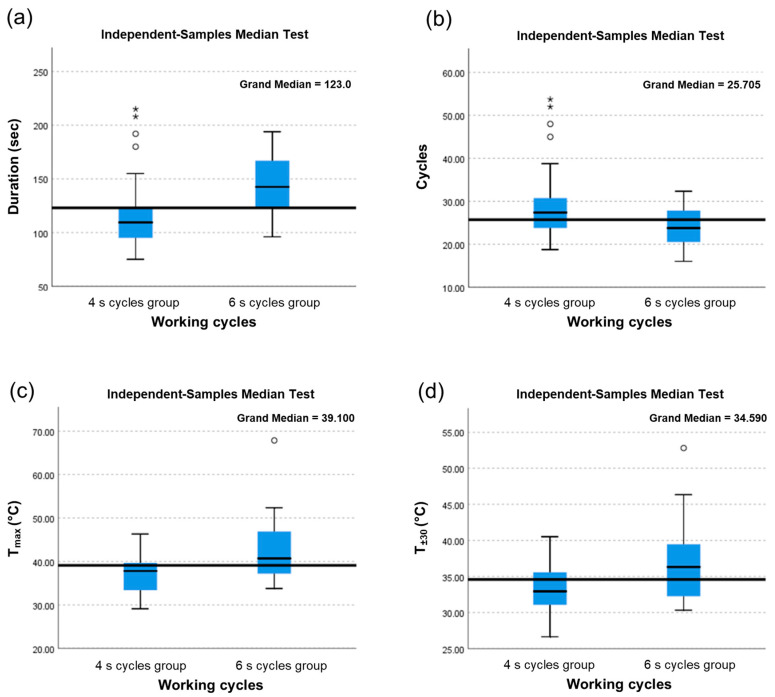
Box plots with median values for each study parameter. (**a**) Duration (s): time necessary to perform each osteotomy to a 10 mm depth; (**b**) Cycles: number of cycles necessary to perform each osteotomy to a 10 mm depth; (**c**) T_max_ (°C): Highest recorded temperature; (**d**) T_±30_ (°C): Arithmetic mean of all temperature data points recorded from 30 s before to 30 s after the highest recorded temperature.

In Figure 3, for each specific variable duration, cycles, T_max_ and T_±30_ overall median value (continuous horizontal line), specific median values from the two experiment’s samples (continuous horizontal line in the boxes), ± standard deviations (topside and low side of the boxes), and ranges are reported. Statistically significant differences between the two experiment’s samples median values are evident in all variables.

## 4. Discussion

Piezoelectric devices are considered a suitable and effective alternative to conventional drilling techniques for implant site preparation. A systematic review with meta-analysis [17] has found that piezoelectric implant site preparation improves implant stability after two and three months from implant placement compared to conventional drilling. The biological reason for this finding may lie in the positive effects of ultrasounds on the early stages of bone healing through the promotion of the expression of bone morphogenetic proteins and, thus, bone remodeling. Overall, these observations further highlight the importance of a proper surgical technique to promote a faster healing process and reduce tissue damage during implant site preparation. Thermal injury during bone instrumentation can impair bone vitality through different mechanisms [18], including necrosis and apoptosis of osteoblasts [19] with subsequent osteonecrosis [20]. A delay in new bone formation has also been reported as a consequence of bone matrix alterations due to overheating [21].

Current knowledge on heat generation and temperature variation in oral bone surgery is mostly based on in vitro studies. On the other hand, heat dissipation, diffusion, and their underlying mechanisms are less understood in vivo, whereas other factors, not fully standardizable, can influence these phenomena. In this scenario, our study has been addressed to maintain both the applied load and handpiece movements in a specific range of values. To this aim, a mechanical positioning device (MPD) was used to standardize these two parameters.

Temperature elevation during conventional and piezoelectric instrumentation is a complex phenomenon encompassing a wide variety of elements. As reported in a previous study [22], these factors can be grouped into three different categories: operator-, bone-, and technique-related factors. If it is possible to reduce the negative effects of operator- and technique-related factors, bone characteristics can influence thermal behavior independently. In fact, differences in cortical/trabecular bone ratio and intrinsic bone mineral density (BMD) can elicit a different thermal response during bone cutting [23]. Conversely, a strictly standardized operative technique and trained operator can minimize temperature elevation, thus reducing the risk of overheating. Technique-related factors are influenced by the technical features of piezoelectric devices and by the shape, design, and irrigation of the ultrasonic tips. Operator-related factors include the applied load and working movements, both of which can be modified and controlled on the basis of an adequate training curve and following specific protocols.

As reported in a previous study [15], a pressure of maximum 150 g results in limited temperature rise, thus the same value has been assumed as the threshold for the current tests. In clinical practice, applied pressure depends strongly on the operator’s experience, assuming that the piezoelectric tip should work, ideally, almost in a passive mode. On the contrary, working movements in piezoelectric implant site preparation have not been investigated in relation to temperature variations, due to the difficulty in standardizing methods. By using the MPD, it is possible to maintain the different phases of the specific movements for piezoelectric implant site preparation, described as alternating quarter-turn rotations. An improvement of movement reproducibility has been obtained by Tang et al. [24] using a robot arm-assisted cutting system. This represents the best approach to standardize variables involved in temperature elevation, however, the semi-automatic motion of MPD could provide data more similar to what can be expected in the clinical practice where the operator’s impact cannot be discarded.

The results of the present study highlighted a statistically significant difference in temperature elevation between the tests performed with 4 s working cycles and those performed with 6 s working cycles. Working cycles of 6 s resulted in significantly higher temperature increase, both for T_max_ and T_±30_. This suggests that prolonged contacts between ultrasonic tip and bone surface determine a greater energy transfer and, consequently, highest temperature values. Moreover, increased contact time also reduces the cooling effect of the irrigation liquid and enhances clogging effect as well, especially with external irrigation system. Therefore, the present results suggest that temperature rise can be effectively minimized by reducing the duration of each cycle. It is interesting to note how a difference of only 2 s can produce completely different thermal effects in bone. On this basis, a marked and uncontrolled temperature increase could be expected with prolonged working cycles (more than 6 s). The importance of the uplift phase (2 s in both groups) to provide an adequate cooling by the irrigation liquid is also evident. Moreover, it must be observed that all the tests of this study were conducted using a pilot ultrasonic tip with an external irrigation and that possible small areas of thermal injury will be removed by the following tips with larger diameters. Therefore, there is all the more reason to consider the subsequent tips (with internal irrigation), and to finalize the implant bed in an equally safe manner.

Another interesting finding concerns the duration of the tests, which was significantly shorter using 4 s working cycles. In other words, less time is necessary to reach the 10 mm cutting depth if 4 s cycles are used. Although the cutting efficiency of ultrasonic devices is not a research topic of the present study, these findings seem to suggest that a greater cutting efficiency can be obtained by reducing the duration of cycles from 6 to 4 s. This could be explained in terms of more effective removal of bone debris from the contact zone, thus avoiding tip clogging and consequent reduction in cutting efficacy. However, further experimental studies are needed to assess the relationship between cutting efficiency and working movement.

Finally, certain measurement variability was found in the tests. It is likely to be due to this intrinsic variability in bone thermal behavior, rather than due to experimental setting limitations, that no statistically significant correlation was found between duration, cycles, and temperature variables. Since bone can be considered a non-homogeneous and anisotropic tissue [25,26], it is the authors’ opinion that anisotropic characteristics can strongly influence the thermal effect of ultrasonic bone cutting, determining variability in measurements, even within the same bone sample. Thus, translation of the present results to the clinical practice should be made with care.

This study has some limitations, mainly related to its in vitro setting. In particular, the use of bovine bone, though considered one of the most representative models, may not precisely resemble all the conditions observed in vivo, due to the lack of vascularization. Future studies should further enhance the level of standardization of the tests in order to correlate multiple variables simultaneously and assess their specific contribution to temperature rise.

## 5. Conclusions

The results of this ex vivo study showed that rapid working cycles of 4 s may significantly limit heat generation during piezoelectric implant site preparation. Most importantly, these results indicate that it is possible to control the operator-related factors that possibly influence temperature elevation in bone surgery by adopting a specific operative protocol. From this perspective, a light pressure (from 100 to 150 g), combined with rapid longitudinal-rotational movements represent the most important factors to consider during piezoelectric implant site preparation. These data confirm those of previous studies in indicating piezoelectric implant site preparation as a safe procedure with respect to temperature elevation.

## Figures and Tables

**Table 1:** Means of the study parameters.

	4 s Cycles Group		6 s Cycles Group		*p* Value ^#^
	Mean	SD	Mean	SD	
**Duration (s)**	119.80	36.4	143.17	29.4	0.002
**Cycles**	29.95	9.1	23.80	4.9	0.005
**T_max_ (°C)**	37.24	4.6	42.44	7.3	0.002
**T_±30_ (°C)**	33.30	3.3	37.01	5.2	0.003

SD, standard deviation. ^#^ Mann–Whitney U test.

**Table 2:** Medians of the study parameters.

	4 s Cycles Group	6 s Cycles Group	*p* Value ^#^
	Median	Median	
**Duration (s)**	108.67	142.50	<0.0001
**Cycles**	27.17	23.75	0.020
**T_max_ (°C)**	37.82	40.71	0.020
**T_±30_ (°C)**	32.94	36.32	0.020

SD, standard deviation. ^#^ Median test.

**Table 3:** Comparison of variances in the two experimental groups.

	F *	*p* Value
**Duration (s)**	0.13	0.72
**Cycles**	4.35	0.04
**T_max_ (°C)**	2.37	0.13
**T_±30_ (°C)**	3.48	0.07

* Equal variances assumed.
